# Role of the Phospholipase C Pathway and Calcium Mobilization in Oxytocin-Induced Contraction of Lacrimal Gland Myoepithelial Cells

**DOI:** 10.1167/iovs.62.14.25

**Published:** 2021-11-23

**Authors:** Angela Gárriz, Salome Aubry, Quentin Wattiaux, Jeffrey Bair, Michael Mariano, Georgios Hatzipetrou, Maytal Bowman, Junji Morokuma, Gustavo Ortiz, Pedram Hamrah, Darlene A. Dartt, Driss Zoukhri

**Affiliations:** 1 Department of Comprehensive Care, Tufts University School of Dental Medicine, Boston, Massachusetts, United States; 2 Schepens Eye Research Institute/Massachusetts Eye and Ear, Department of Ophthalmology, Harvard Medical School, Boston, Massachusetts, United States; 3 Department of Ophthalmology, Tufts University School of Medicine, Boston, Massachusetts, United States

**Keywords:** myoepithelial cells, oxytocin, lacrimal gland, calcium, contraction

## Abstract

**Purpose:**

We reported that oxytocin (OXT), added to freshly prepared lacrimal gland lobules, induced myoepithelial cell (MEC) contraction. In other systems, OXT activates phospholipase C (PLC) generating Inositol 1,4,5-trisphosphate (IP_3_) which increases intracellular calcium concentration ([Ca^2+^]_i_) causing contraction. The aim of the current study was to investigate the role of this pathway in OXT-induced contraction of MEC.

**Methods:**

Tear volume was measured using the cotton thread method. Lacrimal gland MEC were isolated and propagated from α-smooth muscle actin (SMA)-green fluorescent protein (GFP) mice, in which MEC express GFP making them easily identifiable. RNA and protein samples were prepared for RT-PCR and Western blotting for G protein expression. Changes in [Ca^2+^]_i_ were measured in Fura-2 loaded MEC using a ratio imaging system. MEC contraction was monitored in real time and changes in cell size were quantified using ImageJ software.

**Results:**

OXT applied either topically to surgically exposed lacrimal glands or delivered subcutaneously resulted in increased tear volume. OXT stimulated lacrimal gland MEC contraction in a dose-dependent manner, with a maximum response at 10^−7^ M. MEC express the PLC coupling G proteins, Gαq and Gα11, and their activation by OXT resulted in a concentration-dependent increase in [Ca^2+^]_i_ with a maximum response at 10^−6^ M. Furthermore, the activation of the IP_3_ receptor to increase [Ca^2+^]_i_ is crucial for OXT-induced MEC contraction since blocking the IP_3_ receptor with 2-APB completely abrogated this response.

**Conclusions:**

We conclude that OXT uses the PLC/Ca^2+^ pathway to stimulate MEC contraction and increase lacrimal gland secretion.

##  

Myoepithelial cells (MECs) are part of the lacrimal gland secretory apparatus.^1^ MECs create an extensive and functional branching network that surrounds the acinar and ductal epithelial cells separating them from the basement membrane and mesenchymal stromal cells.^2^^–^^5^ MECs express cholinergic muscarinic and purinergic receptors and are therefore thought to be able to respond to neural stimuli and contract to expel lacrimal gland fluid and proteins from the acini and ducts.^6^^,^^7^ These cells express a number of contractile proteins, such as α-smooth muscle actin (SMA) and calponin, as well as epithelial markers, such as keratins (keratin 5 and keratin 14).^5^ Despite their potential crucial role in lacrimal gland secretion, there is a meager number of studies investigating the mechanisms regulating lacrimal gland MEC contraction. In contrast, these cells have been extensively studied in the mammary gland where their contraction is critical for milk ejection and knockout of SMA expression leads to impaired milk secretion.^8^^–^^10^ MEC contraction in the mammary gland is controlled by activation of the oxytocin receptor (OXTR) by oxytocin (OXT).^11^ Most importantly, in studies with OXT and OXTR knock out (KO) mice lactation is impaired, most likely due to loss of the contractile ability of the MEC and diminished exocytosis in epithelial cells.^9^^,^^10^ To the best of our knowledge, no ocular phonotype, such as dry eye disease, was reported in these mice.

OXT was extensively studied for its classical action as a strong uterotonic effect during labor and stimulation of milk ejection during lactation.^9^^,^^10^ However, OXT exerts its effects on a variety of systems, including the male reproductive system and different organs (pancreas, heart, and kidneys, among others).^12^^–^^18^ Many of the observed effects of OXT are achieved by its action on smooth-muscle cells in the target organs.

Phospholipase C-β (PLC-β) catalyzes the hydrolysis of phosphatidylinositol 4,5- biphosphate (PIP_2_) to generate 2 second messenger molecules: inositol 1,4,5- triphosphate (IP_3_) and diacylglycerol (DAG).^19^ IP_3_ mobilizes calcium (Ca^2+^) from intracellular stores and DAG activates protein kinase C (PKC). IP_3_ interacts with IP_3_ receptors (IP_3_Rs) expressed in endoplasmic reticulum membranes to induce release of the sequestered Ca^2+^ into the cytosol.^19^ Released free cytosolic Ca^2+^ then activates, in conjunction with calmodulin, several protein kinases that phosphorylate key proteins involved in smooth muscle cell contraction/relaxation. Both Gαq and Gα11 stimulate PLC-β isoforms with similar efficiency.^20^^,^^21^ The role of the PLC/Ca^2+^ pathway in regulating OXT induced MEC contraction in the mammary gland and smooth muscle cells is well documented.^11^^,^^21^^–^^23^

We recently reported that both human and murine lacrimal gland MEC express the OXTR and that healthy murine lacrimal gland MEC but not chronically inflamed ones, are able to contract in response to OXT stimulation.^24^ As a first step toward studying the impact of OXT on lacrimal gland MEC function, we aimed in the present study to investigate the role of the PLC/ Ca^2+^ pathway in OXT-induced contraction of lacrimal gland MEC.

## Materials and Methods

### Animals

All experiments described herein were performed in accordance with the Association for Research in Vision and Ophthalmology (ARVO) Statement for the Use of Animals in Ophthalmic and Vision Research and were approved by the Tufts Medical Center Animal Care and Use Committee. C57BL/6 and BALB/c mice were purchased from Taconic (Germantown, NY, USA). Mice were maintained in constant temperature rooms with fixed light/dark intervals of 12 hours length and were fed ad libitum. The αSMA‐GFP mice (C57BL6) / *SMA^CreErt2^* strain, described by Yokota et al.^25^ were a kind gift of Dr. Ivo Kalajzic (UConn Health, Farmington, CT, USA) and were used to obtain MECs.^26^ In these mice, the lacrimal gland MEC, which express SMA, are therefore labeled with GFP. SMA-GFP mice were euthanized and the exorbital lacrimal glands were harvested and processed for RNA extraction, total protein extraction, or for MEC isolation and culture, as described below.

### Measurement of Tear Release

Tear release was measured on lightly anesthetized (isoflurane) mice using phenol red impregnated cotton threads (Zone-Quick; Lacrimedics, San Mateo, CA, USA), as previously described.^27^ The threads were held with jeweler forceps and applied to the ocular surface, on both eyes, in the lateral canthus for 10 seconds. Wetting of the thread (which turns red in contact with tears) was measured in millimeters under a dissecting microscope. To stimulate tear release, OXT was either topically applied to surgically exposed exorbital lacrimal glands (measures tear release) or was delivered via subcutaneous (SC) injection (measures tear volume). Carbachol and pilocarpine, two cholinergic muscarinic agonists, were used as positive controls for tear stimulation.

### Myoepithelial Cell Isolation and Culture

Lacrimal glands were removed from 4 to 6 week old SMA-GFP mice and minced into lobules for collagenase digestion using our previously described protocol.^7^ Lacrimal glands were washed in cold DMEM (Dulbecco's Modified Eagle; Gibco, Waltham, MA, USA), gently minced with a scalpel and forceps to prepare 2 to 3 mm lobules and placed in digestion media (1.5 mL/gland of DMEM and 1.65 mg/mL of collagenase type II; Gibco). Samples were then incubated in a shaking water bath (37°C and 100 rpm) for 20 to 30 minutes. At regular 5-minute intervals, lobules were gently pipetted, 10 times, through tips of decreasing diameter. Digested media was filtered through a sterile cell strainer (100 µm Nylon Mesh; Thermo Fisher Scientific, Waltham, MA, USA), the remaining tissue was pushed through the mesh using the pipette tip, and all cells collected washed with 1 to 2 mL DMEM. Cells were then centrifuged at 100 g for 5 minutes, resuspended in complete RPMI-1640 medium (Roswell Park Memorial Institute) supplemented with 10% fetal bovine serum, 2 mM L-glutamine, and 100 µg/mL penicillin-streptomycin; Gibco) and centrifuged again at 100 g for 5 minutes. Pelleted cells were resuspended in 10 mL complete RPMI media, plated in 75 mm culture dishes (VWR, Radnor, PA, USA), and placed in a 37°C incubator (5% CO_2_). Confluent cells were trypsinized using TrypLE Express (trypsin replacement; Invitrogen, Carlsbad, CA, USA).

### Lacrimal Gland Imaging by Intravital Multiphoton Microscopy

Animal preparation was performed as previously described.^28^ Briefly, mice were anesthetized by intraperitoneal injection of ketamine (100 mg/kg)/xylazine (20 mg/kg) cocktail, which results in up to 75 minutes of deep anesthesia. Prior to the incision, hair between the eye and ear (approximately 10 mm wide) was carefully removed using Nair hair removal lotion (Naircare, Princeton, NJ, USA), followed by a single dose injection of 30 µL local analgesic (0.75% Bupivacaine HCl). An approximately 5 mm cutaneous incision was made 2 mm away from the eye and 3 mm away from the ear to expose the exorbital lacrimal gland. Careful removal of the soft tissues around the lacrimal gland was performed to expose it without damaging the blood vessels. In order to stabilize the lacrimal gland during imaging, a wooden spatula was placed underneath it. Afterward, 5 to 10 µL of PBS were carefully injected into the lacrimal gland capsule (connective tissue that surrounds the lobes of the lacrimal gland). This created a separation between the capsule and the lobes of the gland to enable removal of the capsule without damaging the underlying lobes. The mouse body temperature was maintained between 35 and 37°C using a disposable hand warmer (HotHands; HearMax, Dalton, GA, USA). Intra vital-multiphoton microscopy (IV-MPM) was performed using an Ultima Multiphoton Microscope System (Bruker, Fitchburg, WI, USA) equipped with 2 MaiTai Ti/Sapphire DeepSee lasers (Newport Spectra-Physics, Irvine, CA, USA). The laser power was set at 95, and the photomultiplier tube gain (PMTs) was set at 650 for all channels. Using a 20x-1.0 NA (Olympus XLUMPLFLN; Olympus, Tokyo, Japan) water immersion objective, scans of the lacrimal gland were taken with 512 × 512 resolution and 2-fold line averaging. GenTeal ophthalmic lubricant gel (Alcon, Fort Worth, TX, USA) was applied onto the glands and the incision to prevent desiccation during imaging. In order to stain the blood vessels, mice were retro-orbital injected with 100 µL of a 1:10 dilution of QTRACKER Qdot 655-Vascular Label (Cat # Q21021MP; Thermo Fisher Scientific, Waltham, MA, USA).

### Image Analysis

In order to examine the GFP+ cells within the lacrimal gland, 4D movies and 3D images were generated by importing the image stacks to the Imaris software (Bitplane, Zurich, Switzerland) as previously described.^28^^,^^29^ The number of GFP-labeled MEC was counted semi-automatically in XYZ positions using the 3D rendering function of Imaris software*.*

### Measurement of MEC Contraction

Between 4500 and 5000 MEC were seeded overnight into a 24-well plate. Video recording was performed using a digital camera (Vanguard USB 5 Megapixel ISH500 Digital Camera; Vanguard, Hanson, MA, USA) mounted on an inverted light microscope (Vanguard; Precise Instrument). Still images and lapse time videos were captured using the manufacturer TCapture software (version 3.9). Cells were stimulated with OXT (10^−8^, 10^−7^, or 10^−6^ M) and viewed by video for 20 minutes. Still images were taken at 0 and 20 minutes after OXT stimulation for image analyses. At least 10 random cells from each well and each condition were used for image analyses, using ImageJ software (Image J 1.53a; National Institutes of Health, Bethesda, MD, USA). The perimeter of each cell was calculated before and after OXT stimulation and the decrease in cell size after OXT stimulation was expressed in a percentage.

### RNA Extraction and RT-PCR Analysis

RNA from lysed MEC and lacrimal gland tissue samples was extracted using the RNeasy isolation mini kit (Qiagen, Valencia, MA, USA) following the manufacturer's protocol. The RNA concentration was measured using a NanoDrop 1000 (Thermo Fisher Scientific). Purified total RNA (50–250 ng) was used for reverse transcription and PCR amplification using the OneStep RT-PCR Kit (Qiagen, Valencia, MA, USA) with primers (GαQ: Fw 5′-CGTCCTGTTCGCTTTAGAGG-3′ Rv 5′-CTGTCCATGGTGGTGTCAAG-3′; Gα11: Fw 5′-GTACCC-GTTTGACCTGGAGA-3′Rv 5′-CTCCACCAGGACTTGGTCAT-3′; and GAPDH: Fw 5′-GGTGAAGGTCGGTGTGAACG-3′ Rv 5′-CTCGCTCCTGGAAGATGGTG-3′) designed using NCBI/ Primer-BLAST in a 2720 Thermal Cycler (Applied Biosystems, Foster City, CA, USA). The reverse transcription reaction and the cycling conditions were conducted according to the manufacturer's instructions. Next, the amplification products were separated by electrophoresis on a 2% agarose gel and visualized by UV light after SYBR safe DNA gel stain (Invitrogen, Carlsbad, CA, USA).

### SDS-PAGE and Western Blotting

Lacrimal gland MEC grown in 6-well plates were homogenized in 0.2 mL ice-cold radio-immunoprecipitation assay (RIPA) buffer (10 mM Tris-HCl pH 7.4, 150 mM NaCl, 1 mM EDTA, 1% Triton X-100, 0.1% sodium deoxycholate, 10 mM DTT, and 0.1% SDS supplemented with a protease inhibitor cocktail; Sigma-Aldrich, St. Louis, MO, USA). Proteins were separated by SDS-PAGE on NuPage 4 to 12% Bis-Tris gels in MOPS-SDS buffer (Invitrogen) and transferred to polyvinylidene difluoride (PVDF) membranes. Non-specific binding was blocked using Intercept blocking buffer (LI-COR Biosciences, Lincoln, NE) for 1 hour at room temperature. Membranes were then incubated overnight at 4°C with either rabbit polyclonal anti-Gαq (1:500; Abcam, Cambridge, MA, USA) or mouse monoclonal anti-Gα11 (1:200; Santa Cruz Biotechnology, Dallas, TX, USA) primary antibodies diluted in blocking buffer + 0.1% Tween 20 (to reduce nonspecific binding). Following 3 washes with Tris-buffered saline + tween-20 (TBS; 50 mM Tris-HCl, 150 mM NaCl, and 0.1% tween-20; pH 7.6) membranes were incubated for 1 hour at room temperature with the appropriate secondary antibodies, a goat anti-rabbit IRDye 800 (LI-COR Biosciences) or a goat anti-mouse IRDye 680 (LI-COR Biosciences), diluted 1:5000 followed by detection on a LI-COR ODYSSEY CLx Infrared Imager.

### Measurement of Intracellular [Ca^2+^]

MEC were seeded on 35 mm glass bottom petri dishes (Cellvis, Sunnyvale, CA, USA) at a density of 5000 cells per dish. They were loaded with the intracellular Ca^2+^ indicator fura-2/AM (0.5 µM) for 1 hour in the dark. Then cells were stimulated with OXT (10^−8^, 10^−7^, and 10^−6^ M). In selected experiments, cells were preincubated for 20 minutes with the IP_3_ receptor antagonist, 2-aminoethoxydiphenyl borate (2-APB; 10^−4^ M; Tocris Bioscience, Bristol, UK) before addition of OXT. UTP (10^−5^ M) was used as a positive control in each experiment. Changes in intracellular [Ca^2+^] were recorded using a ratio imaging system (InCyt Im2; Intracellular Imaging, Cincinnati, OH, USA).^7^ As fura-2 binds to free calcium in the cell, its peak absorption wavelength changes from 380 nm (unbound) to 340 nm (bound), whereas the emission wavelength remains at 510 nm. The 510 nm emissions are captured by the camera as black and white images and the ratio of emission intensity excited by 340 nm and 380 nm light is recorded. These ratios are compared to a standard curve derived from standard calcium solutions to determine ion concentration. The use of ratios ensures that measurements are not changed by variable dye concentration or cell thickness.

### Data Presentation and Statistical Analysis

Where appropriate, data are expressed as mean ± SEM. The data were statistically analyzed using either unpaired Student's *t*-test or 1-way analysis of variance (ANOVA) followed by Dunnett's multiple comparisons test using GraphPad Prism version 8.2. The *P* values below 0.05 were considered statistically significant.

## Results

### OXT Increases Tear Volume

**Figure 1.**
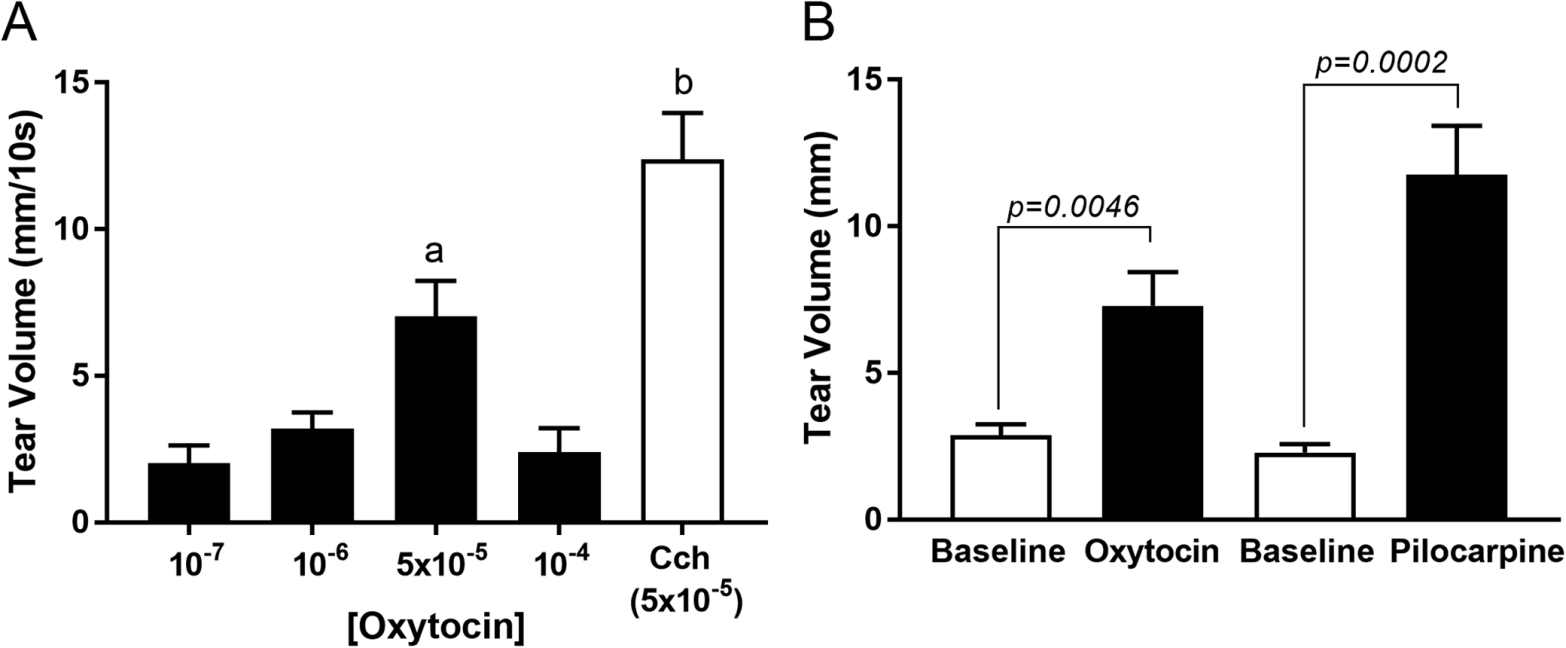
Measurements of tear volume using phenol red impregnated cotton threads. (**A**) Changes in tear volume were observed after increasing concentrations of oxytocin (OXT; 10^−7^, 10^−6^, 5 × 10^−5^, or 10^−4^ M) were topically applied to surgically exposed exorbital lacrimal glands. Tear volume was significantly increased at OXT concentration of 5 × 10^−5^ compared to the other OXT concentrations. Carbachol, a cholinergic muscarinic agonist (Cch, 5 × 10^−5^ M), was used as a positive control and significantly increased tear volume compared with all OXT concentration treatments. Statistically significant differences are represented by letters “a” and “b,” *P* < 0.01, *n* = 8 to 9 for OXT and *n* = 4 for Cch. (**B**) Measurements of tear volume before (baseline) and after OXT (2.2 IU per mouse) or pilocarpine (3.5 mg/kg) were subcutaneously injected. There was a significant increase in tear volume after OXT injection (*P* = 0.0046) and pilocarpine injection (*P* = 0.0002) compared with each baseline, *n* = 6.

We reported that OXT, added to freshly prepared lacrimal gland lobules, induced a decrease in acini size indicative of stimulation of MEC contraction.^24^ To determine if this hormone is capable of increasing tear output, we measured tear volume, using phenol red impregnated cotton threads, after OXT was either topically applied to surgically exposed exorbital lacrimal glands for tear release or was subcutaneously (SC) injected for tear volume. Carbachol and pilocarpine, two cholinergic muscarinic agonists known to stimulate lacrimal gland secretion, were used as positive controls. Previous studies reported the expression of M_3_ muscarinic receptors by lacrimal gland MEC.^6^ As shown in Figure 1A, topical application of OXT increased tear release with a maximum 7.03 ± 1.21 mm obtained at a 5 × 10^−6^ M. The positive control, carbachol at 5 × 10^−6^ M, increased tear volume to 12.38 ± 1.60 mm. OXT delivered SC increased tear release from a baseline value of 2.88 ± 0.39 mm to 7.29 ± 1.16 mm (Fig. 1B). For comparison, pilocarpine increased tear release from a baseline value of 2.29 ± 0.28 mm to 11.77 ± 1.67 mm (see Fig. 1B).

### Isolation and Propagation of Lacrimal Gland MEC

**Figure 2.**
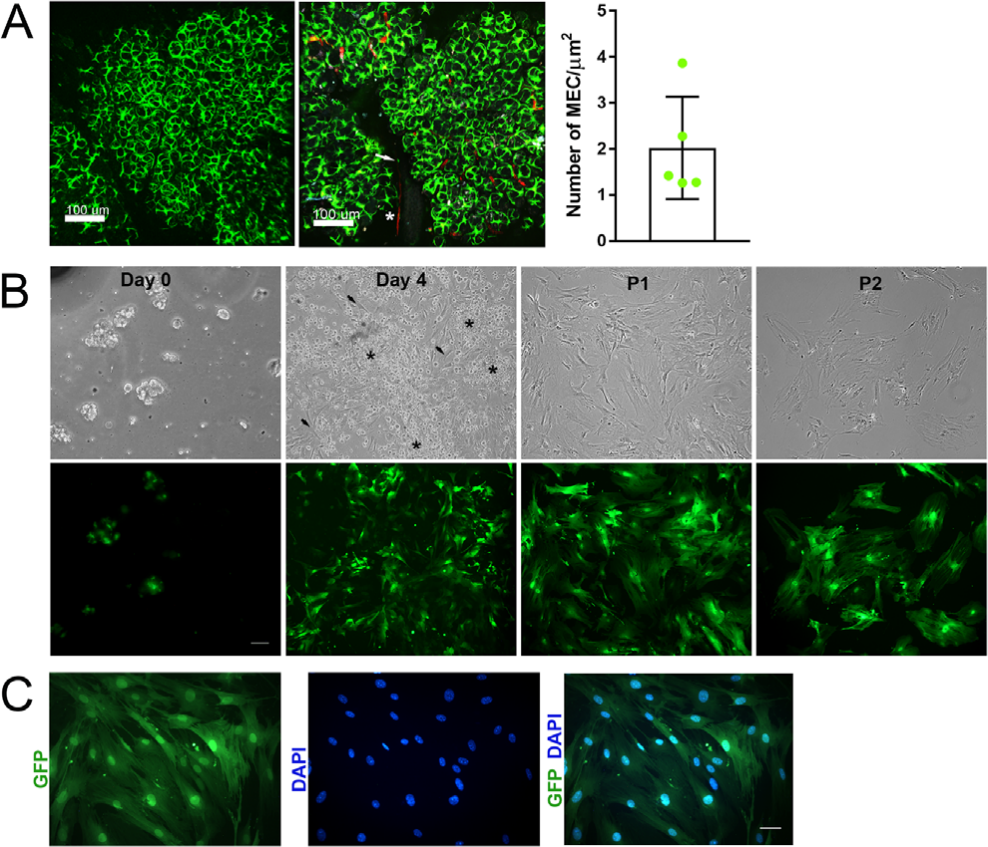
(**A**) In vivo images of lacrimal glands from αSMA‐GFP mice, (C57BL6)/*SMA^CreErt2^* strain, obtained using multiphoton microscopy. The left image shows MEC-GFP+ in lacrimal gland lobules surrounding acini. The image on the right shows MEC-GFP+ and blood vessels (red) stained with QDots. Arrow indicate the potential presence of GFP+ pericytes surrounding the blood vessel wall (star). The graph depicts the number of MEC/µm^2^ counted semi-automatically in XYZ positions using the 3D rendering function of Imaris software. Data are means ± S.D., *n* = 5. (**B**) Pictures at days 0 and 4, Passage 1 and Passage 2 of MEC isolated from the lacrimal gland of αSMA‐GFP mice (C57BL6)/*SMA^CreErt2^* strain. Black arrows indicate the cells attaching to the plate and the stars indicate floating cells. Scale bar = 100 µm. (**C**) Images of P2 GFP-MEC counterstained with DAPI showing that all DAPI+ cells are also GFP+. Scale bar = 100 µm.

We used SMA-GFP mice to isolate lacrimal gland MEC. IV-MPM of the lacrimal glands showed the extensive expression of GFP by the MEC (Fig. 2A, Supplementary Video S1). The number of GFP-labeled MEC was counted semi-automatically using the 3D rendering function of Imaris software and report a mean ± SD of 2.02 ± 1.11 cells/µm^2^ (see Fig. 2A).

Because GFP expression was under the SMA promoter and other cells beside the MEC, such as pericytes, also express SMA, we labeled blood vessels with QTRACKER Qdot 655-Vascular Label and performed IV-MPM. As shown in Figure 2A (Supplementary Video S2), some pericytes do express GFP but their number appears relatively low compared to that of GFP-MEC positive cells.

Next, lacrimal glands from SMA-GFP mice were used to isolate and propagate MEC using our published protocol.^7^^,^^30^ As shown in Figure 2B, MEC-GFP positive cells can be seen surrounding GFP-negative acinar epithelial cell clusters in freshly digested lacrimal gland lobules (day 0). After 2 to 4 days in culture, GFP-positive cells started to adhere to the plastic culture dish (see Fig. 2B) and by 7 to 10 days, they became the predominant cell type. As stated above, SMA is also expressed by pericytes and therefore these can be present in our culture system. As reported with rat lacrimal gland MEC, murine lacrimal gland MEC can be passaged up to three times.^7^ Passage 1 (P1) and P2 cells shown in Figure 2B indicate that after passage, GFP-positive MECs are the only cell type present. To rule out the presence of non-GFP expressing cells in our culture system, cell nuclei were stained with DAPI. As shown in Figure 2C, all DAPI positive nuclei are GFP positive. It should be noted that GFP(-) fibroblasts, without being apparent in early preparations, may become activated by 2D culture, causing them to begin to express SMA and GFP, a possibility that cannot be ruled out.

### OXT Stimulates Lacrimal Gland MEC Contraction

**Figure 3.**
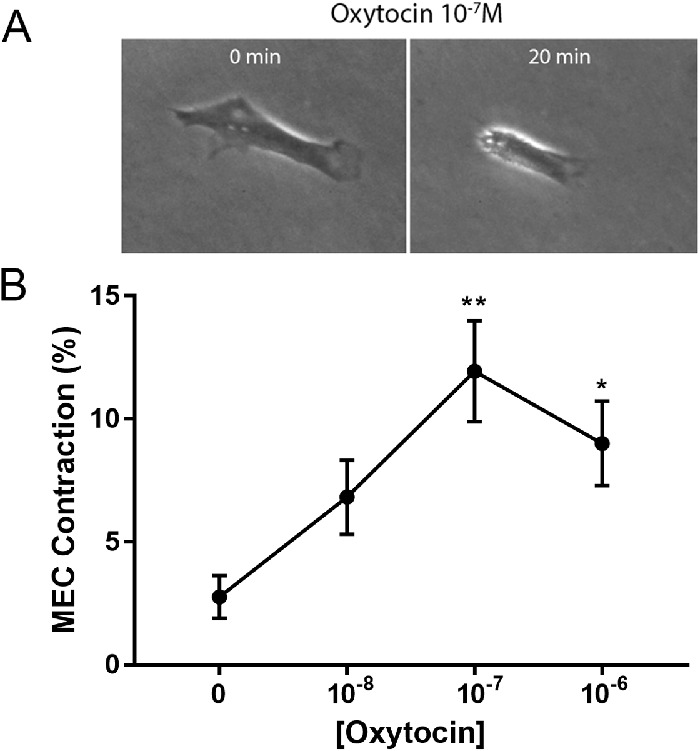
Lacrimal gland myoepithelial cells (MECs) contraction measurements after oxytocin (OXT) stimulation, performed by a real time monitor system. (**A**) Example of lacrimal gland MEC contraction before (0 min) and after (20 min) of OXT stimulation (10^−7^ M). (**B**) Measurement of lacrimal gland MEC contraction. The average of cell area measured in 10 cells per photograph was measured before and after OXT stimulation representing the lacrimal gland MEC contraction expressed as a percentage (%). A significant increase of MEC contraction after OXT stimulation at concentrations of 10^−7^ M and 10^−6^ M was observed, compared to 0. Statistically significant differences are represented by: * = *P* < 0.01 and ** = *P* < 0.001, *n* = 3 to 4.

We used lacrimal gland MEC in P2 or P3 to measure contraction following OXT stimulation using ImageJ software. Cells were plated at very low density (approximately 5000 cells) in 24-well plates and imaged under an inverted microscope equipped with a digital camera. We used a seeding density that allowed proper identification of individual cell borders during image analysis and a time in culture that made it easier for cells to “contract” (detach from the plastic culture dish and reduce in size). Changes in cell size, either spontaneously or in response to OXT stimulation, were measured using still images taken before and 20 minutes after stimulation using ImageJ software. Figure 3A and Supplementary Video S3 shows an example of change in cell shape following a 20-minute stimulation with 10^−7^ M OXT. In the absence of stimulation, lacrimal gland MEC displayed a spontaneous change in cell shape of about 2.5% (Fig. 3B). Addition of OXT for 20 minutes induced a concentration-dependent decrease in lacrimal gland MEC size with a maximum of 11.5% at 10^−7^ M (see Fig. 3B, Supplementary Videos S3, S4). It should be noted that OXT induced MEC contraction can be detected 5 minutes post addition but reaches a maximum by 20 minutes (Supplementary Video S5).

#### Lacrimal Gland MEC Express PLC Coupling G-Proteins

**Figure 4.**
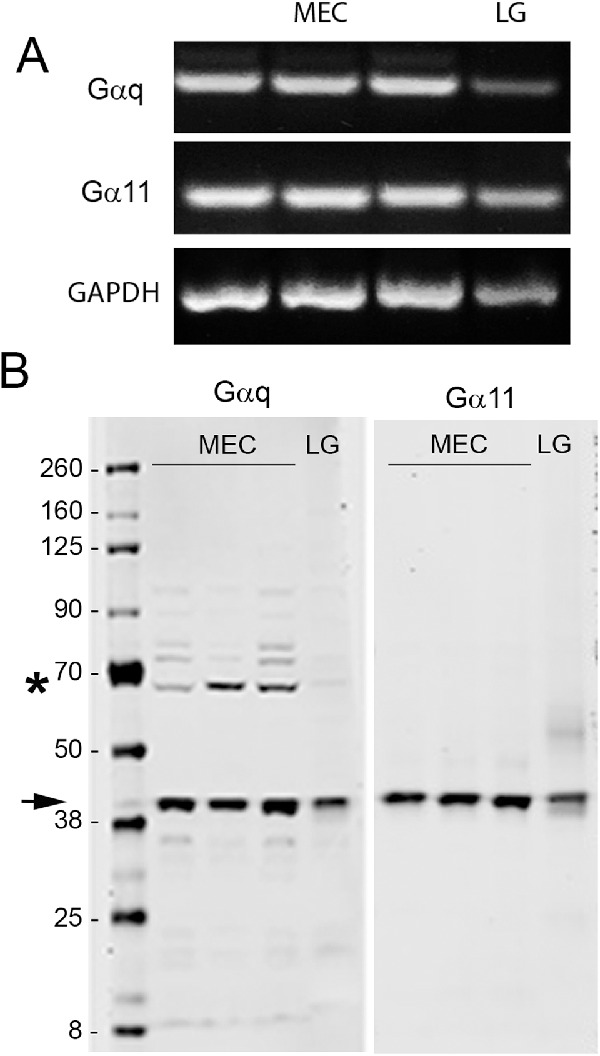
The mRNA and protein expression of G proteins subunits αq and α11 from lysed myoepithelial cells (MEC) and lacrimal gland (LG) tissue samples. (**A**) The mRNA expression of Gαq, Gα11, and GAPDH which was used as a housekeeping gene. (**B**) Gαq and Gα11 protein expression in a SDS Page/Western blotting. Black arrow indicates Gαq and Gα11 migrating at the predicted molecular weight. Black star indicates additional bands detected in the Gαq blot.

As OXT is known to use the PLC/Ca^2+^ pathway to induce MEC contraction in other tissues, we determined if lacrimal gland MEC express the PLC-coupling G proteins, Gαq and Gα11, using RT-PCR and Western blotting. Total RNA and cell lysate from whole lacrimal gland were used as a positive control. As shown in Figure 4, expression of both G proteins can be detected both at the mRNA (see Fig. 4A) as well as the protein (see Fig. 4B) level in both MEC and lacrimal gland samples. Both Gαq and Gα11 proteins migrated with the predicted molecular weight of approximately 40 KDa for Gαq and approximately 43 KDa for Gα11 (see Fig. 4, arrow).^31^^–^^33^ Additional nonspecific bands were detected in the Gαq blot, especially in the MEC samples (see Fig. 4, star).

#### OXT Stimulation Increases Intracellular Calcium Concentration in Lacrimal Gland MEC

**Figure 5.**
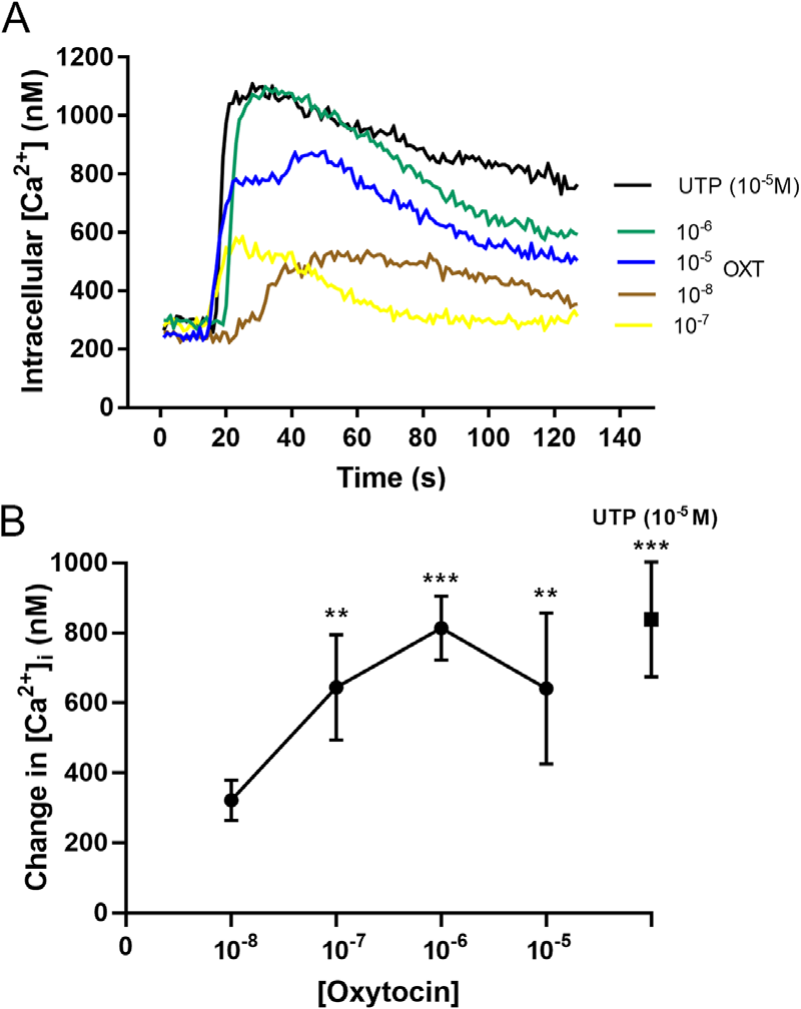
Measurement of change in intracellular Ca^2+^ concentrations ([Ca^2+^]i nM) in lacrimal gland myoepithelial cells (MEC) during stimulation with oxytocin (OXT 10^−8^ to 10^−5^ M). (**A**) Representative Ca^2+^ traces over time elicited by increasing concentrations of OXT. (**B**) Change in ([Ca^2+^]_i_ (nM) after incubation of lacrimal gland MEC with OXT. A concentration-dependent increase of [Ca^2+^]_i_ was observed and it was significantly different in OXT 10^−7^, 10^−6^, and 10^−5^ when they were compared to 0. UTP (10^−5^ M) was used as a positive control. Statistically significant differences are represented by: *P < 0.05; **P < 0.001, and ***P < 0.0001, *n* = 5.

To determine if OXT activates PLC, we monitored changes in intracellular calcium concentration ([Ca^2+^]_i_). Ca^2+^ is mobilized from intracellular stores by IP_3_, generated following hydrolysis of PIP_2_ by activated PLC and binding to its receptor, IP_3_R. Figure 5A depicts the time-dependent increase in [Ca^2+^]_i_ elicited by OXT. Incubation of lacrimal gland MEC with OXT elicited a concentration-dependent increase in [Ca^2+^]_i_ with a maximum at 10^−6^ M (Fig. 5B). The response elicited by 10^−6^ M (813.5 ± 90.1 nM) OXT was comparable to that triggered by UTP (10^−5^ M, 838.4 ± 82.0 nM), a P2Y2 agonist known to increase [Ca^2+^]_i_ in lacrimal gland MEC.^7^

#### OXT Stimulation of Lacrimal Gland MEC is Dependent on IP_3_-Induced Ca^2+^ Release

**Figure 6.**
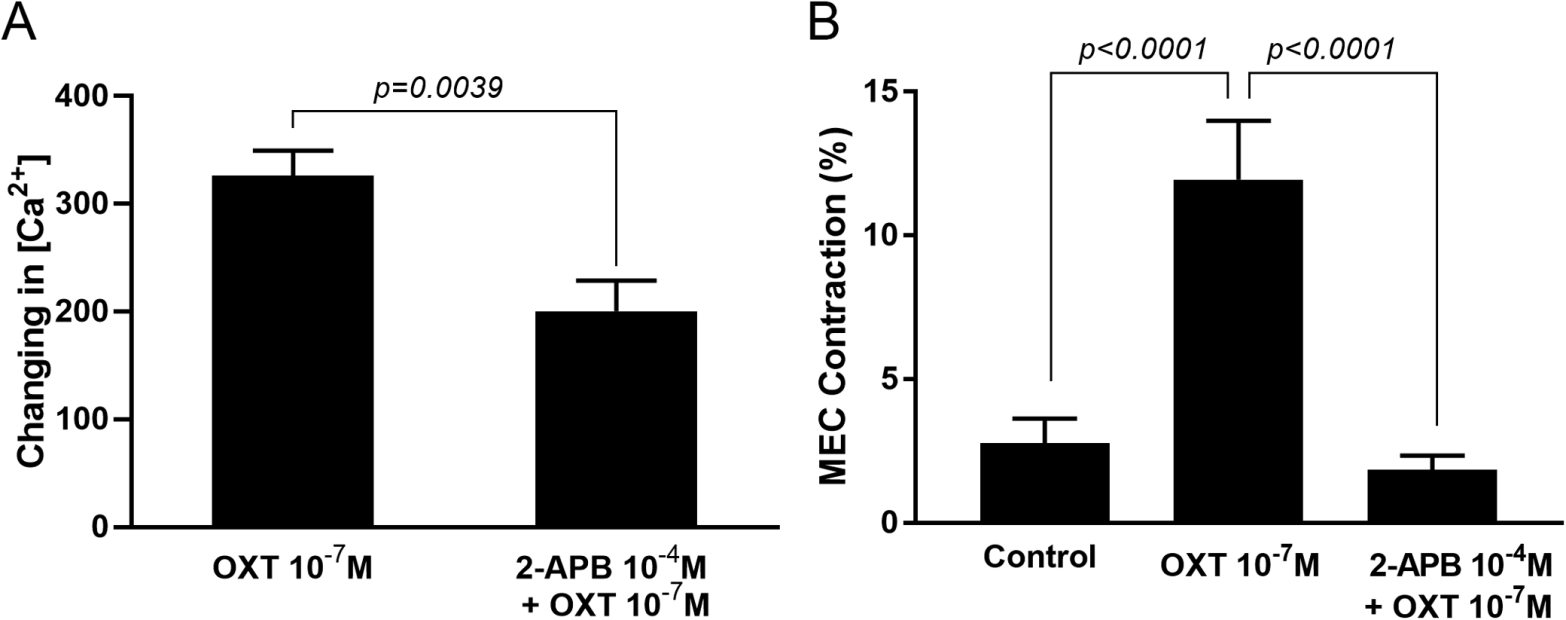
Effect of Inositol 1,4,5-trisphosphate (IP_3_) receptor antagonist, 2-aminoethoxydiphenyl borate (2-APB), which blocks IP_3_-mediated Ca^2+^ release from the endoplasmic reticulum on myoepithelial cell (MEC) contraction. (**A**) Changes in intracellular [Ca^2+^] in lacrimal gland MEC stimulated with 10^−7^ M of oxytocin (OXT) alone or after addition of 2-APB (10^−4^ M). There was a statistically significant decrease in intracellular [Ca^2+^] in lacrimal gland MEC treated with 2-APB (*P* = 0.0039, *n* = 3). (**B**) Lacrimal gland MEC contraction after 20 minutes of addition of vehicle (control), 10^−7^ M of OXT (OXT 10^−7^ M), and 10^−7^ M of OXT after addition of 2-APB (10^−4^ M). There was a statistically significant decrease in the control and 2-APB 10^−4^ M + OXT 10^−7^ M compared with OXT 10^−7^ M (*P* < 0.0001, *n* = 3).

To determine the role of PLC-induced Ca^2+^ release in OXT-stimulated lacrimal gland MEC contraction, we used an IP_3_ receptor antagonist, 2-aminoethoxydiphenyl borate (2-APB), which blocks IP_3_-mediated Ca^2+^ release from the endoplasmic reticulum. First, we tested the effect of 2-APB on OXT-induced increase in [Ca^2+^]_i_. OXT (10^−7^ M) increased [Ca^2+^]_i_ to 326.3 ± 23.1 nM (Fig. 6A). Preincubation of lacrimal gland MEC with 2-APB (10^−4^ M) significantly decreased the OXT-induced mobilization of intracellular Ca^2+^ to 200.4 ± 28.6 nM. OXT (10^−7^ M) increased lacrimal gland MEC contraction by 11.95 % compared to basal 2.77% (Fig. 6B). Treatment with 2-APB (10^−4^ M) before addition of OXT (10^−7^ M) completely inhibited OXT-induced lacrimal gland MEC contraction.

OXT stimulation of lacrimal gland MEC is dependent on IP_3_-induced Ca^2+^ release.

## Discussion

Lacrimal gland MEC are known to express the OXTR and OXT to induce MEC contraction in lacrimal gland lobules.^24^ In the current study, we show that OXT, applied either topically to surgically exposed lacrimal gland or SC increases tear volume as measured by the phenol red thread assay. We also showed that lacrimal gland MEC can be isolated and propagated from mouse lacrimal gland using the same protocol we used for isolating these cells from rat lacrimal glands. Using these cells, we showed that OXT stimulates lacrimal gland MEC contraction in a dose-dependent manner and that PLC-induced increase in [Ca^2+^]_i_ is crucial for this response, in agreement with reports from other cells/tissues.^34^^,^^35^

The OXTR belongs to a small receptor family of classical G-protein coupled receptors (GPCRs) also containing the structurally related arginine-vasopressin receptors (V1aR, V1bR, and V2R).^12^ Depending on the type of the coupling G protein (Gαq/11, Gαs, or Gαi/o), the activation generates different second messengers and thus leads to distinct responses. For example, OXT mediated contraction in myometrial cells involves the activation of a calcium-dependent pathway mediated by Gαq/11 and the decrease in cAMP levels mediated by Gαi.^34^^,^^35^ In human embryonic kidney HEK293 cells, OXT stimulates cell growth via Gαq/11 coupling, whereas Gαi coupling inhibits it.^36^^,^^37^ Finally, in immortalized olfactory neurons, OXTR coupling to Gαq/11 decreases inward rectifying potassium (K+) currents in a subset of cells, whereas, in a different subpopulation, OXTR coupling to Gαi increases them.^38^ OXT-stimulated PLC via Gαq and Gα11 has been well described in rat and human myometrial cells.^20^^,^^21^

In the present study, we showed that MEC express the PLC coupling G proteins, Gαq and Gα11, and that their activation resulted in a concentration-dependent increase in [Ca^2+^]_i_. We also reported that OXT stimulated MEC contraction and that activation of the IP_3_ receptor to increase [Ca^2+^]_i_ is crucial for OXT-induced MEC contraction because blocking the IP_3_ receptor with 2-APB completely abrogated OXT-induced MEC contraction. The 2-APB was the first membrane-permeant modulator of IP_3_ receptor described and it is still considered a useful tool to investigate the physiological role of IP_3_ in different cell types.^39^ It should be noted, however, that several studies have reported that 2-APB have effects on Ca^2+^ mobilization not related to inhibition of IP_3_-induced Ca^2+^ release, such as blockade of store-operated Ca^2+^ entry pathways.^40^^–^^42^ A role for the store operated Ca^2+^ channel, Orai1, in mammary gland MEC contraction and lactation has been elegantly demonstrated by Davis et al.^43^ Future studies should address the role of Ca^2+^ channels in lacrimal gland MEC contraction and tearing.

Stevenson et al.^44^ using transgenic mice that express the fast, ultrasensitive Ca^2+^ indicator (GCaMP) reported that, in contrast to mammary MEC, they could not detect OXT-mediated increase in [Ca^2+^]_i_ or contractile response in MEC from the lacrimal gland. In contrast, Satoh et al.^45^ using Fura-2 and Indo-1 as calcium indicators coupled with digital imaging analyses reported that cholinergic stimulation of lacrimal gland MEC induced increases in [Ca^2+^]_i_ concomitant with contraction. More recently, Jin et al.,^46^ using a mouse line expressing Yellow Cameleon 3.60 (a genetically encoded Ca^2+^ indicator) and intravital two-photon imaging, also reported increased [Ca^2+^]_i_ in lacrimal gland MEC in response to cholinergic stimulation. The use of different mouse cell lines and imaging techniques could explain the discrepancies between these studies.

Our findings indirectly implied that OXT-induced increase in [Ca^2+^]_i_ and MEC contraction could be responsible for the observed increase in tear volume. In support of this conclusion, Jin et al.^46^ reported correlation between the agonist evoked increases in [Ca^2+^]_i_ in lacrimal gland MECs to stimulation of tear release. More importantly, they showed that post-ganglionic denervation of the lacrimal gland (to mimic dry eye disease) led to decreased cholinergic-induced Ca^2+^ mobilization in MEC that correlated with decreased tear release. Based on these comparative studies (intravital Ca^2+^ signaling in MEC and tear release in healthy compared with denervated glands), the authors concluded that Ca^2+^ signaling in lacrimal gland MECs participate in tear-secretory function.

Coupling of the OXTR via Gαs activates adenylate cyclase to increase intracellular cyclic adenosine monophosphate (cAMP) levels whereas coupling via Gαi/o has an opposite effect on adenylate cyclase and decreases cytosolic cAMP concentration. Several reports showed that activation of this pathway counterbalances the Gαq/11-dependent effect and self-limits the OXT-induced cell contractile responses.^47^ Studies are currently underway to determine if OXT alters cAMP levels in lacrimal gland MECs and if activation of this pathway leads to MEC contraction and/or attenuates the OXTR-Gαq/11-dependent stimulatory pathway described in the present report.

Although OXT is released in response to a variety of stimuli (e.g. suckling, parturition, and stress) from the posterior pituitary into the systemic circulation that lead to an intranuclear release of OXT. Sometimes, OXT is also synthesized in peripheral tissues, such as uterus, placenta, amnion, testis, and heart.^12^ The source(s) and mechanisms of release of OXT in the lacrimal gland remain to be investigated.

In summary, our data showed that OXT stimulates lacrimal gland MEC contraction and increases aqueous tear volume; and that the PLC/calcium pathway plays a major role in this response.

## Supplementary Material

Supplement 1

Supplement 2

Supplement 3

Supplement 4

Supplement 5

## Supplementary Material

**Supplementary Videos S1 and S2.** Imaging of lacrimal glands from αSMA‐GFP mice (C57BL6)/*SMA^CreErt2^* strain obtained using multiphoton microscopy. In this video GFP+ cells are lacrimal gland MEC that can be observed surrounding acini in several lobules. SMA is the main marker for MEC, but it can also be present in other cell types such as pericytes. In Video S2, blood vessels were stained with QTRACKER Qdot 655-Vascular Label and are visualized in red. GFP+ pericytes can be seen surrounding the blood vessels. The 4D movies were generated using the Imaris Software (Bitplane, Zurich, Switzerland).

**Supplementary Video S3.** Time-lapse video of a cultured lacrimal gland MEC stimulated with oxytocin (OXT, 10^−7^ M). The cell was recorded using Tcapture software (version 3.9) for a total of 20 minutes after addition of OXT.

**Supplementary Video S4.** Time-lapse video of cultured lacrimal gland MECs stimulated with oxytocin (OXT, 10^−7^ M). The cell was recorded for a total of 20 minutes after addition of OXT using Tcapture software (version 3.9).

**Supplementary Video S5.** Time-lapse video of a cultured lacrimal gland MEC stimulated with oxytocin (OXT, 10^−7^ M) for 5 minutes recorded using Tcapture software (version 3.9). The arrow points to the cell edge that is seen contracting in response to OXT stimulation.

## References

[1] Dartt DA. Neural regulation of lacrimal gland secretory processes: relevance in dry eye diseases. *Prog Retin Eye Res*. 2009; 28: 155–177. 19376264 10.1016/j.preteyeres.2009.04.003 PMC3652637

[2] Beha G, Sarli G, Brunetti B, Sassi F, Ferrara D, Benazzi C. Morphology of the myoepithelial cell: immunohistochemical characterization from resting to motile phase. *The Scientific World Journal*. 2012; 2012: 252034. 22919300 10.1100/2012/252034 PMC3420080

[3] Leeson TS, Leeson CR. Myoepithelial cells in the exorbital lacrimal and parotid glands of the rat in frozen-etched replicas. *The Am J Anatomy*. 1971; 132: 133–145. 10.1002/aja.1001320202 5112466

[4] Wang YL, Tan Y, Satoh Y, Ono K. Morphological changes of myoepithelial cells of mouse lacrimal glands during postnatal development. *Histol Histopathol*. 1995; 10: 821–827. 8574002

[5] Makarenkova HP, Dartt DA. Myoepithelial Cells: Their Origin and Function in Lacrimal Gland Morphogenesis, Homeostasis, and Repair. *Curr Mol Biol Rep*. 2015; 1: 115–123. 26688786 10.1007/s40610-015-0020-4 PMC4683023

[6] Lemullois M, Rossignol B, Mauduit P. Immunolocalization of myoepithelial cells in isolated acini of rat exorbital lacrimal gland: cellular distribution of muscarinic receptors. *Biol Cell*. 1996; 86: 175–181. 8893507 10.1016/0248-4900(96)84782-4

[7] Ohtomo K, Shatos MA, Vrouvlianis J, Li D, Hodges RR, Dartt DA. Increase of intracellular Ca2+ by purinergic receptors in cultured rat lacrimal gland myoepithelial cells. *Invest Ophthalmol Vis Sci*. 2011; 52: 9503–9515. 22039237 10.1167/iovs.11-7809 PMC3341118

[8] Haaksma CJ, Schwartz RJ, Tomasek JJ. Myoepithelial Cell Contraction and Milk Ejection Are Impaired in Mammary Glands of Mice Lacking Smooth Muscle Alpha-Actin. *Biology of Reproduction*. 2011; 85: 13–21. 21368298 10.1095/biolreprod.110.090639 PMC3123380

[9] Nishimori K, Young LJ, Guo Q, Wang Z, Insel TR, Matzuk MM. Oxytocin is required for nursing but is not essential for parturition or reproductive behavior. *Proc Natl Acad Sci USA*. 1996; 93: 11699–11704. 8876199 10.1073/pnas.93.21.11699 PMC38121

[10] Takayanagi Y, Yoshida M, Bielsky IF, et al. Pervasive social deficits, but normal parturition, in oxytocin receptor-deficient mice. *Proc Natl Acad Sci USA*. 2005; 102: 16096–16101. 16249339 10.1073/pnas.0505312102 PMC1276060

[11] Nakano H, Furuya K, Yamagishi S. Synergistic effects of ATP on oxytocin-induced intracellular Ca2+ response in mouse mammary myoepithelial cells. *Pflugers Arch*. 2001; 442: 57–63. 11374069 10.1007/s004240100521

[12] Gimpl G, Fahrenholz F. The oxytocin receptor system: structure, function, and regulation. *Physiol Rev*. 2001; 81: 629–683. 11274341 10.1152/physrev.2001.81.2.629

[13] Altura BM, Altura BT. Actions of vasopressin, oxytocin, and synthetic analogs on vascular smooth muscle. *Fed Proc*. 1984; 43: 80–86. 6690341

[14] Bodanszky M, Sharaf H, Roy JB, Said SI. Contractile activity of vasotocin, oxytocin, and vasopressin on mammalian prostate. *Eur J Pharmacol*. 1992; 216: 311–313. 1397015 10.1016/0014-2999(92)90376-f

[15] Frayne J, Nicholson HD. Localization of oxytocin receptors in the human and macaque monkey male reproductive tracts: evidence for a physiological role of oxytocin in the male. *Mol Hum Reprod*. 1998; 4: 527–532. 9665335 10.1093/molehr/4.6.527

[16] Amico JA, Finn FM, Haldar J. Oxytocin and vasopressin are present in human and rat pancreas. *Am J Med Sci*. 1988; 296: 303–307. 3195625 10.1097/00000441-198811000-00003

[17] Petersson M, Alster P, Lundeberg T, Uvnas-Moberg K. Oxytocin causes a long-term decrease of blood pressure in female and male rats. *Physiol Behav*. 1996; 60: 1311–1315. 8916187 10.1016/s0031-9384(96)00261-2

[18] Favaretto AL, Ballejo GO, Albuquerque-Araujo WI, Gutkowska J, Antunes-Rodrigues J, McCann SM. Oxytocin releases atrial natriuretic peptide from rat atria in vitro that exerts negative inotropic and chronotropic action. *Peptides*. 1997; 18: 1377–1381. 9392839 10.1016/s0196-9781(97)00209-x

[19] Berridge MJ. Inositol trisphosphate and calcium signalling. *Nature (London)*. 1993; 361: 315–325. 8381210 10.1038/361315a0

[20] Strakova Z, Soloff MS. Coupling of oxytocin receptor to G proteins in rat myometrium during labor: Gi receptor interaction. *Am J Physiol*. 1997; 272: E870–E876. 9176188 10.1152/ajpendo.1997.272.5.E870

[21] Ku CY, Qian A, Wen Y, Anwer K, Sanborn BM. Oxytocin stimulates myometrial guanosine triphosphatase and phospholipase-C activities via coupling to G alpha q/11. *Endocrinology*. 1995; 136: 1509–1515. 7895660 10.1210/endo.136.4.7895660

[22] Raymond K, Cagnet S, Kreft M, Janssen H, Sonnenberg A, Glukhova MA. Control of mammary myoepithelial cell contractile function by alpha3beta1 integrin signalling. *EMBO J*. 2011; 30: 1896–1906. 21487391 10.1038/emboj.2011.113 PMC3098485

[23] Bird GS, Aziz O, Lievremont JP, Wedel BJ, Trebak M, Vazquez G, Putney JW Jr. Mechanisms of phospholipase C-regulated calcium entry. *Curr Mol Med*. 2004; 4: 291–301. 15101686 10.2174/1566524043360681

[24] Hawley D, Tang X, Zyrianova T, et al. Myoepithelial cell-driven acini contraction in response to oxytocin receptor stimulation is impaired in lacrimal glands of Sjogren's syndrome animal models. *Sci Rep*. 2018; 8: 9919. 29967327 10.1038/s41598-018-28227-x PMC6028591

[25] Yokota T, Kawakami Y, Nagai Y, et al. Bone marrow lacks a transplantable progenitor for smooth muscle type alpha-actin-expressing cells. *Stem Cells*. 2006; 24: 13–22. 16099999 10.1634/stemcells.2004-0346

[26] Kalajzic Z, Li H, Wang LP, et al. Use of an alpha-smooth muscle actin GFP reporter to identify an osteoprogenitor population. *Bone*. 2008; 43: 501–510. 18571490 10.1016/j.bone.2008.04.023 PMC2614133

[27] Zoukhri D, Macari E, Kublin CL. A single injection of interleukin-1 induces reversible aqueous-tear deficiency, lacrimal gland inflammation, and acinar and ductal cell proliferation. *Exp Eye Res*. 2007; 84: 894–904. 17362931 10.1016/j.exer.2007.01.015 PMC3234164

[28] Ortiz G, Chao C, Jamali A, et al. Effect of Dry Eye Disease on the Kinetics of Lacrimal Gland Dendritic Cells as Visualized by Intravital Multi-Photon Microscopy. *Front Immunol*. 2020; 11: 1713. 32903439 10.3389/fimmu.2020.01713 PMC7434984

[29] Seyed-Razavi Y, Lopez MJ, Mantopoulos D, et al. Kinetics of corneal leukocytes by intravital multiphoton microscopy. *FASEB J*. 2019; 33: 2199–2211. 30226811 10.1096/fj.201800684RR PMC6338630

[30] Garcia-Posadas L, Hodges RR, Utheim TP, Olstad OK, Delcroix V, Makarenkova HP, Dartt DA. Lacrimal Gland Myoepithelial Cells Are Altered in a Mouse Model of Dry Eye Disease. *Am J Pathol*. 2020; 190: 2067–2079. 32679229 10.1016/j.ajpath.2020.06.013 PMC7520664

[31] Blank JL, Ross AH, Exton JH. Purification and characterization of two G-proteins that activate the beta 1 isozyme of phosphoinositide-specific phospholipase C. Identification as members of the Gq class. *J Biol Chem*. 1991; 266: 18206–18216. 1655741

[32] Mullaney I, Mitchell FM, McCallum JF, Buckley NJ, Milligan G. The human muscarinic M1 acetylcholine receptor, when express in CHO cells, activates and downregulates both Gq alpha and G11 alpha equally and non-selectively. *FEBS Lett*. 1993; 324: 241–245. 8508928 10.1016/0014-5793(93)81401-k

[33] Meneray MA, Fields TY, Bennett DJ. Gs and Gq/11 couple vasoactive intestinal peptide and cholinergic stimulation to lacrimal secretion. *Invest Ophthalmol Vis Sci*. 1997; 38: 1261–1270. 9152245

[34] Sanborn BM. Hormones and calcium: mechanisms controlling uterine smooth muscle contractile activity. The Litchfield Lecture. *Exp Physiol*. 2001; 86: 223–237. 11429639 10.1113/eph8602179

[35] Zhou XB, Lutz S, Steffens F, Korth M, Wieland T. Oxytocin receptors differentially signal via Gq and Gi proteins in pregnant and nonpregnant rat uterine myocytes: implications for myometrial contractility. *Mol Endocrinol*. 2007; 21: 740–752. 17170070 10.1210/me.2006-0220

[36] Busnelli M, Sauliere A, Manning M, Bouvier M, Gales C, Chini B. Functional selective oxytocin-derived agonists discriminate between individual G protein family subtypes. *J Biol Chem*. 2012; 287: 3617–3629. 22069312 10.1074/jbc.M111.277178 PMC3281696

[37] Rimoldi V, Reversi A, Taverna E, et al. Oxytocin receptor elicits different EGFR/MAPK activation patterns depending on its localization in caveolin-1 enriched domains. *Oncogene*. 2003; 22: 6054–6060. 12955084 10.1038/sj.onc.1206612

[38] Gravati M, Busnelli M, Bulgheroni E, et al. Dual modulation of inward rectifier potassium currents in olfactory neuronal cells by promiscuous G protein coupling of the oxytocin receptor. *J Neurochem*. 2010; 114: 1424–1435. 20557424 10.1111/j.1471-4159.2010.06861.x

[39] Maruyama T, Kanaji T, Nakade S, Kanno T, Mikoshiba K. 2APB, 2-aminoethoxydiphenyl borate, a membrane-penetrable modulator of Ins(1,4,5)P3-induced Ca2+ release. *J Biochem*. 1997; 122: 498–505. 9348075 10.1093/oxfordjournals.jbchem.a021780

[40] Bootman MD, Collins TJ, Mackenzie L, Roderick HL, Berridge MJ, Peppiatt CM. 2-aminoethoxydiphenyl borate (2-APB) is a reliable blocker of store-operated Ca2+ entry but an inconsistent inhibitor of InsP3-induced Ca2+ release. *FASEB J*. 2002; 16: 1145–1150. 12153982 10.1096/fj.02-0037rev

[41] Peppiatt CM, Collins TJ, Mackenzie L, et al . 2-Aminoethoxydiphenyl borate (2-APB) antagonises inositol 1,4,5-trisphosphate-induced calcium release, inhibits calcium pumps and has a use-dependent and slowly reversible action on store-operated calcium entry channels. *Cell Calcium*. 2003; 34: 97–108. 12767897 10.1016/s0143-4160(03)00026-5

[42] Park MK, Lee KK, Uhm DY. Slow depletion of endoplasmic reticulum Ca(2+) stores and block of store-operated Ca(2+) channels by 2-aminoethoxydiphenyl borate in mouse pancreatic acinar cells. *Naunyn Schmiedebergs Arch Pharmacol*. 2002; 365: 399–405. 12012026 10.1007/s00210-002-0535-0

[43] Davis FM, Janoshazi A, Janardhan KS, et al . Essential role of Orai1 store-operated calcium channels in lactation. *Proc Natl Acad Sci USA*. 2015; 112: 5827–5832. 25902527 10.1073/pnas.1502264112 PMC4426473

[44] Stevenson AJ, Vanwalleghem G, Stewart TA, et al. Multiscale imaging of basal cell dynamics in the functionally mature mammary gland. *Proc Natl Acad Sci USA*. 2020; 117: 26822–26832. 33033227 10.1073/pnas.2016905117 PMC7604439

[45] Satoh Y, Sano K, Habara Y, Kanno T. Effects of carbachol and catecholamines on ultrastructure and intracellular calcium-ion dynamics of acinar and myoepithelial cells of lacrimal glands. *Cell Tissue Res*. 1997; 289: 473–485. 9232826 10.1007/s004410050893

[46] Jin K, Imada T, Nakamura S, et al. Intravital Two-photon Imaging of Ca(2+) signaling in Secretory Organs of Yellow Cameleon Transgenic Mice. *Sci Rep*. 2018; 8: 15880. 30367106 10.1038/s41598-018-34347-1 PMC6203801

[47] Liu H, Gruber CW, Alewood PF, Moller A, Muttenthaler M. The oxytocin receptor signalling system and breast cancer: a critical review. *Oncogene*. 2020; 39: 5917–5932. 32782397 10.1038/s41388-020-01415-8 PMC7483001

